# Necrotizing Fasciitis of the Lower Extremity Treated With Continuous Local Antibiotic Perfusion

**DOI:** 10.7759/cureus.66865

**Published:** 2024-08-14

**Authors:** Nana Tomaru, Hideharu Nakamura, Takaya Makiguchi, Yumi Yamada, Satoshi Yokoo

**Affiliations:** 1 Department of Plastic Surgery, National Hospital Organization (NHO) Takasaki General Medical Center, Takasaki, JPN; 2 Department of Oral and Maxillofacial Surgery and Plastic Surgery, Gunma University Graduate School of Medicine, Maebashi, JPN

**Keywords:** negative pressure wound therapy, gentamicin, continuous local antibiotics perfusion, intra-soft tissue antibiotics perfusion, necrotizing fasciitis

## Abstract

Necrotizing fasciitis (NF) is a severe soft tissue infection that can spread rapidly throughout the body, often resulting in fatal outcomes. Negative pressure wound therapy (NPWT) enhances wound healing by applying local negative pressure, and its effectiveness in managing NF has been documented. However, NPWT creates a semi-closed environment at the wound site, posing a risk of infection. Continuous local antibiotic perfusion (CLAP) is an innovative approach that uses localized infusion to deliver high concentrations of antibiotics continuously to the affected area via NPWT. CLAP includes intramedullary antibiotic perfusion (iMAP), which involves the intrathecal administration of antimicrobials, and intra-soft tissue antibiotic perfusion (iSAP), which targets the soft tissue. Previous studies have highlighted the efficacy of CLAP in controlling infections in both bone and soft tissue. Here, we present two cases of lower extremity NF treated with iSAP. In both cases, effective infection control and accelerated wound healing were achieved, leading to wound closure. These outcomes suggest that iSAP is a useful treatment modality for NF that offers a reduced treatment period and minimizes the procedural burden on patients.

## Introduction

Necrotizing fasciitis (NF) is a severe soft tissue infection that can spread rapidly within the skin, subcutaneous fat, fascia, and muscle tissues. Early diagnosis, aggressive debridement, and systemic broad-spectrum antibiotics are required for effective treatment [1]. Although rare, NF has a high mortality that is reported to range from 20% to 35.7% [2-5]. Traditionally, following debridement, wounds often remain open and are managed with ointments and wound dressings until closure is achieved through skin grafting or flap surgery.

Negative pressure wound therapy (NPWT) promotes wound healing by applying local negative pressure and has demonstrated efficacy for various wound types [6-8]. Recently, NPWT has emerged as an effective post-debridement wound management strategy for NF [9-11]. However, this method creates a semi-closed environment within the wound site, which increases the risk of infection [12]. NPWT is generally contraindicated in the presence of residual necrotic tissue, and its use presupposes the removal of necrotic tissue and control of infection within the wound. NPWT with instillation and dwell time (NPWTi-d), which incorporates intermittent wound irrigation with physiological saline into NPWT, has been reported to facilitate early NPWT initiation and promote wound healing [13]. However, saline lavage alone may not suffice for infection control in high-risk wounds.

In continuous local antibiotic perfusion (CLAP), antibiotics are locally infused at high concentrations for continuous delivery to the affected area using NPWT. CLAP includes intramedullary antibiotic perfusion (iMAP), with the intrathecal administration of antimicrobials, and intra-soft tissue antibiotic perfusion (iSAP), in which soft tissues are targeted. Previous studies have highlighted the efficacy of CLAP for controlling infections in bone and soft tissue [14-16]. Several studies have demonstrated the benefits of NPWT and NPWTi-d for NF, but research on the use of iSAP for this condition remains limited [9-11]. Here, we present two cases of lower extremity NF that were treated with iSAP and had favorable outcomes.

## Case presentation

Method

Following debridement for NF, the wounds were left open and iSAP was initiated after the necrotic tissue was sufficiently reduced. In both cases, a V.A.C.® Ulta (Solventum Corp., Maplewood, MN, USA) was used as the NPWT device. V.A.C. Veraflo Cleanse® Choice™ dressing (Solventum Corp.) was applied to the wound surface and maintained at a negative pressure of -75 mmHg. Gentamicin was selected as the antimicrobial agent for iSAP and was administered continuously as a 60 mg solution in 50 ml saline at a rate of 2 ml/hour using a syringe pump. A nasogastric tube (Atom Nutritional Catheter, Atom Medical Corp., Japan) was used for drug delivery, with the inlet and suction paths strategically positioned diagonally to ensure optimal distribution of the medication across the entire wound surface.

Case 1

**Figure 1:**
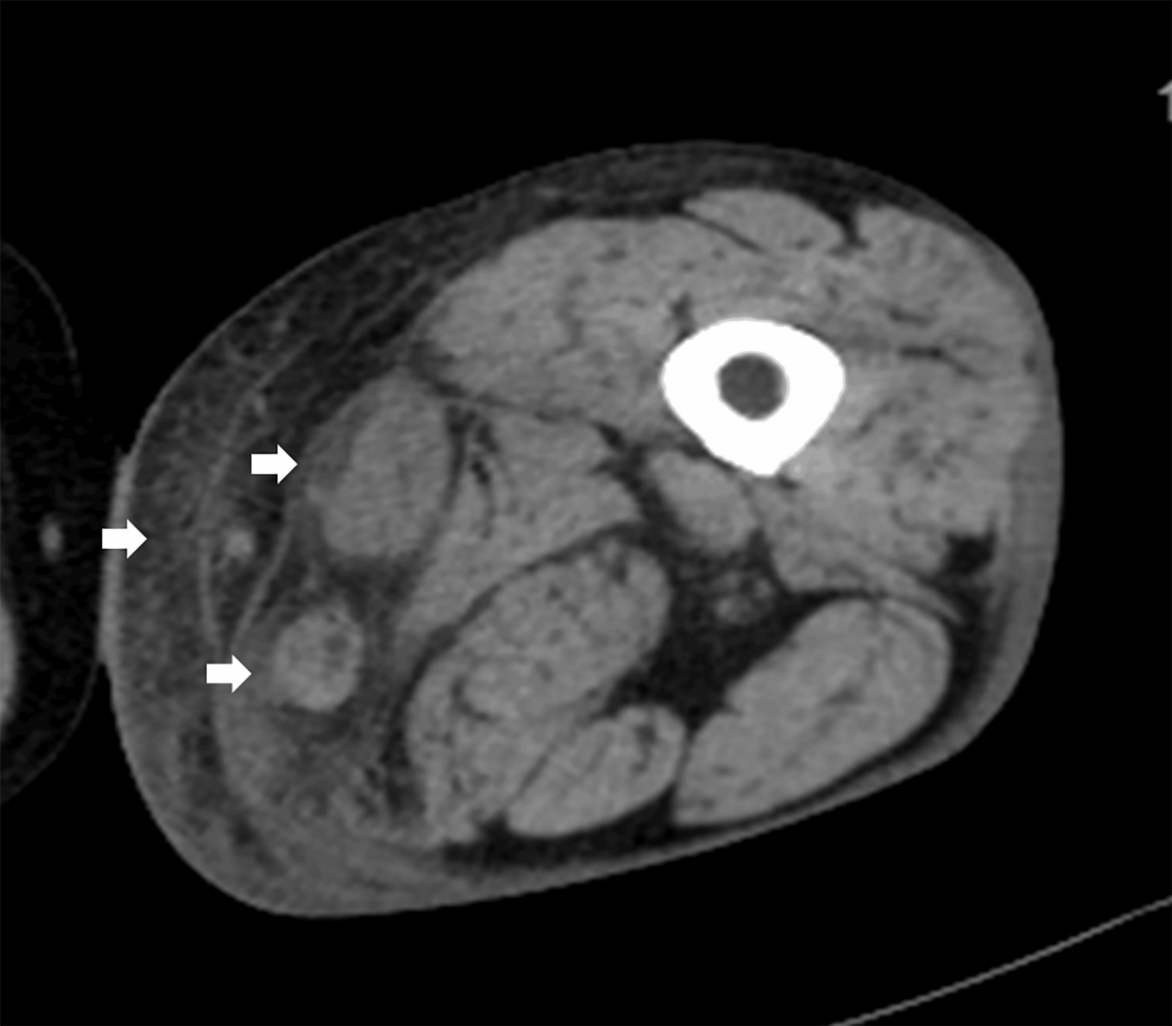
CT findings CT findings showing an increased density of adipose tissue on the medial aspect of the left thigh, with edema noted in the sartorius and gracilis muscles (white arrows). CT: computed tomography

The patient was a 58-year-old female with diabetes who was urgently transported to our hospital due to swelling and pain in the left lower limb. Upon arrival, her vital signs were as follows: body temperature (BT) of 38.5°C, blood pressure (BP) of 190/104 mmHg, heart rate (HR) of 111 beats/min, and oxygen saturation (SpO2) of 95% on room air. Significant swelling and pain were observed around the left knee. Computed tomography (CT) revealed increased adipose tissue density and edema in the sartorius and gracilis muscles of the left thigh (Figure 1). Laboratory tests indicated a white blood cell (WBC) count of 126,000/μL, C-reactive protein (CRP) 28.37 mg/dL, hemoglobin (Hb) 12.5 g/dL, sodium (Na) 133.7 mmol/L, creatinine (Cre) 0.56 mg/dL, glucose (Glu) 431 mg/dL, and hemoglobin A1c (HbA1c) 15.6%. The laboratory risk indicator for NF (LRINEC) score was 7, indicating a high risk of NF.

**Figure 2:**
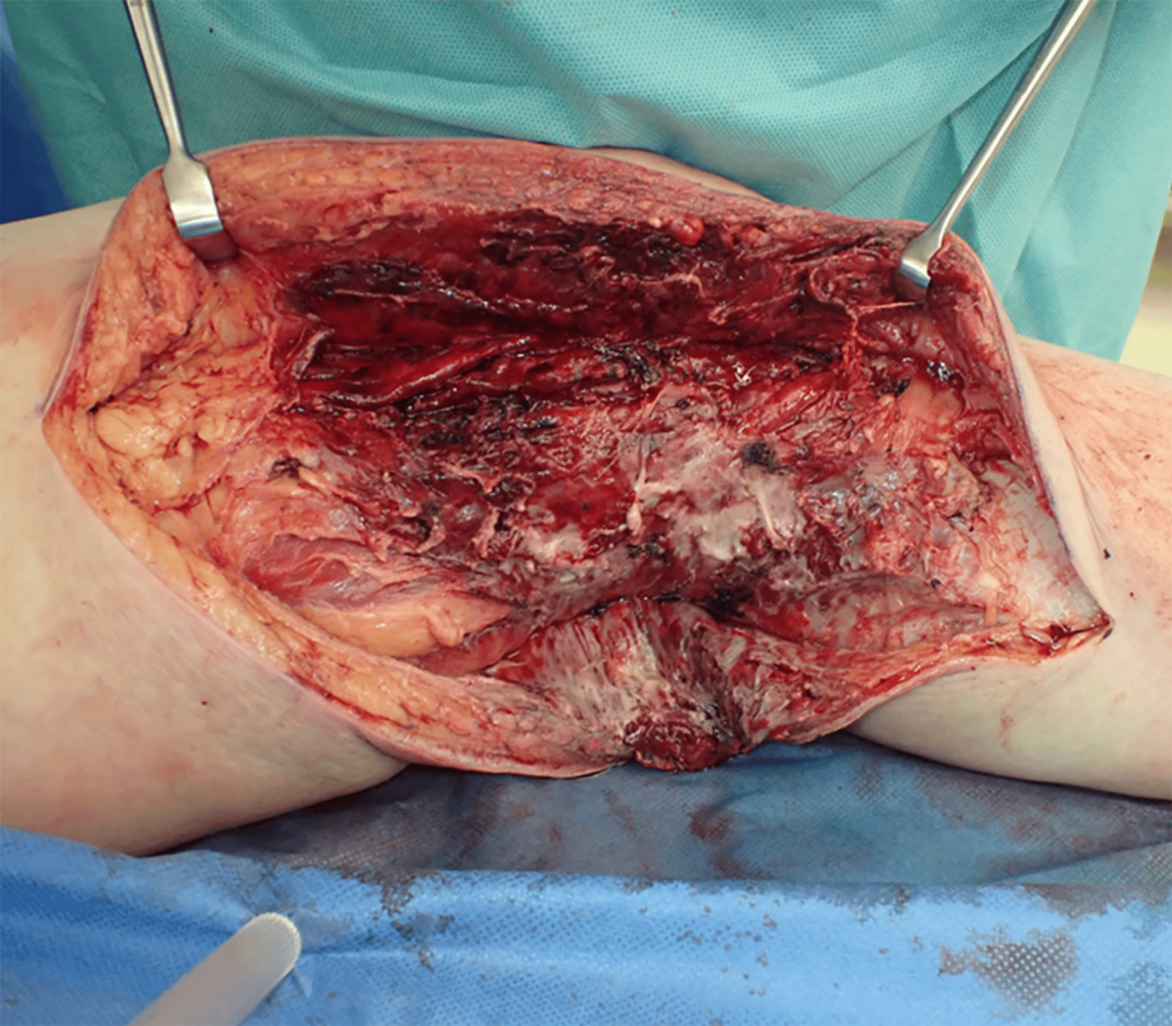
Intraoperative findings Intraoperative findings showing extensive necrosis extending from the subcutaneous tissue to the deep fascia in the medial thigh.

After the diagnosis of NF, debridement under general anesthesia was performed on the same day, and this procedure revealed extensive necrosis from the subcutaneous tissue to the deep fascia in the medial thigh (Figure 2). Bacterial cultures were obtained from the same area, and antibiotics were initiated with meropenem (MEPM) 3 g/day and clindamycin (CLDM) 2400 mg/day. The wound remained open and daily irrigation was performed. Detection of methicillin-sensitive *Staphylococcus aureus* in the wound culture led to the de-escalation of antibiotics to cefazolin (CEZ) 3 g/day and CLDM 2400 mg/day starting from the postoperative day (POD) 4. Antibiotics were discontinued on POD 16 after the confirmation of a significant reduction of necrotic tissue.

**Figure 3:**
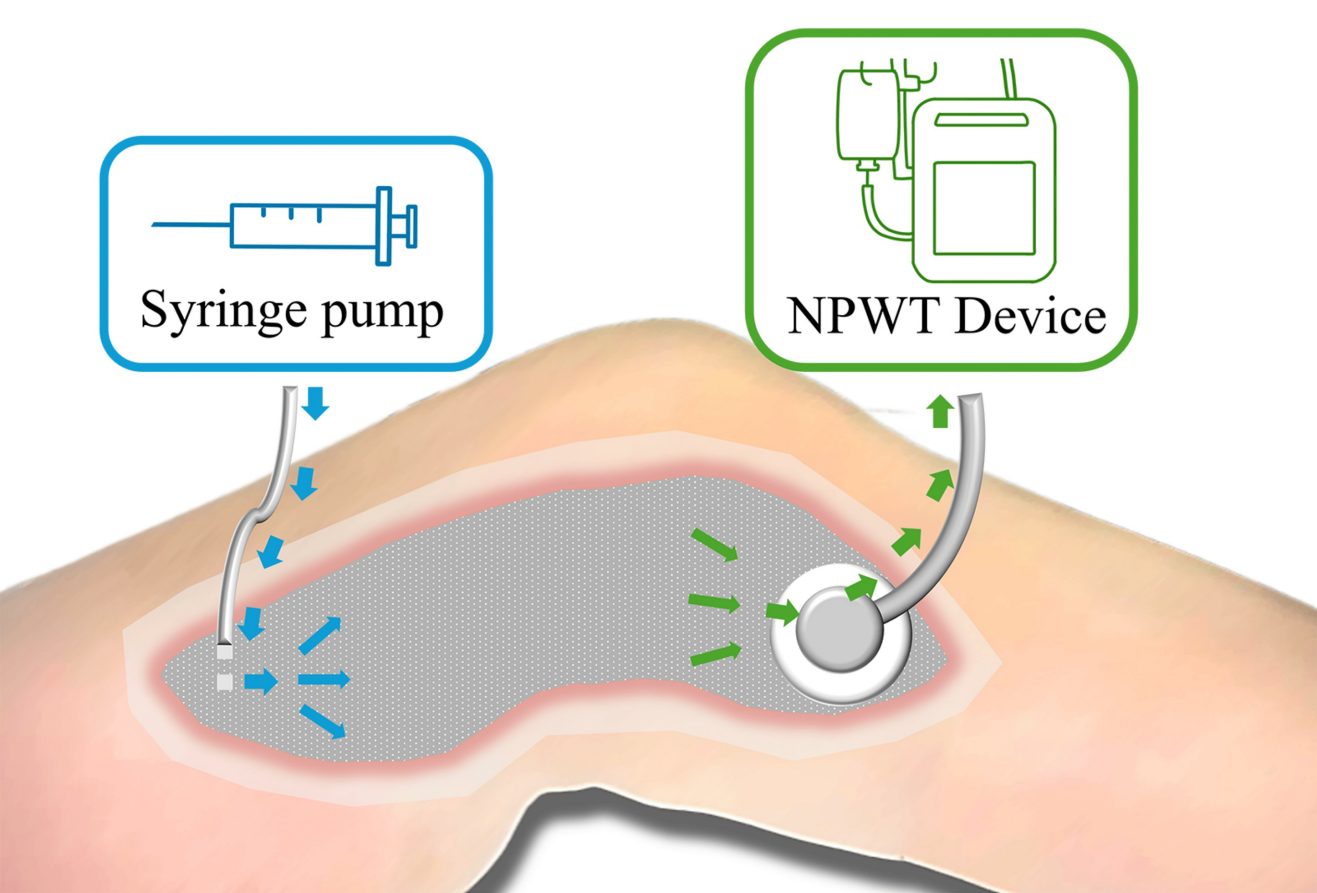
Schema of iSAP Schema of iSAP after debridement for necrotizing fasciitis. A gentamicin injection tube was positioned under the proximal thigh skin (blue arrows), and a suction tube was attached distally to the wound (green arrows). iSAP: intra-soft tissue antibiotic perfusion; NPWT: negative pressure wound therapy

iSAP was started on POD 17 with a gentamicin injection tube placed under the skin of the proximal thigh and a suction tube attached distally (Figure 3). Foam exchange was performed twice weekly. There was no recurrence of inflammation or adverse events related to gentamicin post-iSAP application.

**Figure 4:**
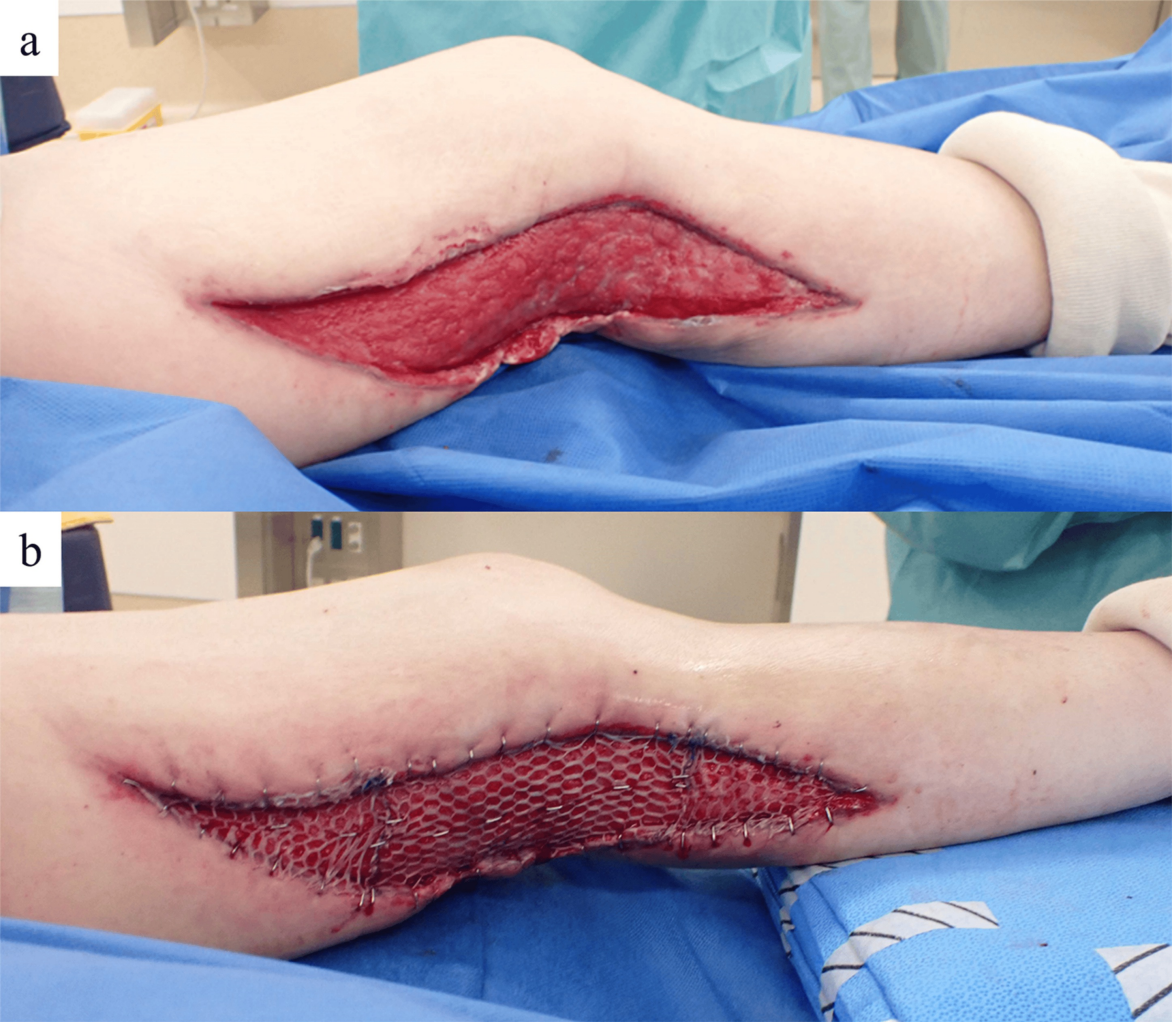
Wound findings two weeks after the iSAP application Two weeks after the iSAP application, the necrotic tissue had disappeared, and good granulation tissue growth was observed (a). Wound closure was then performed using a split-thickness skin graft (b). iSAP: intra-soft tissue antibiotic perfusion

**Figure 5:**
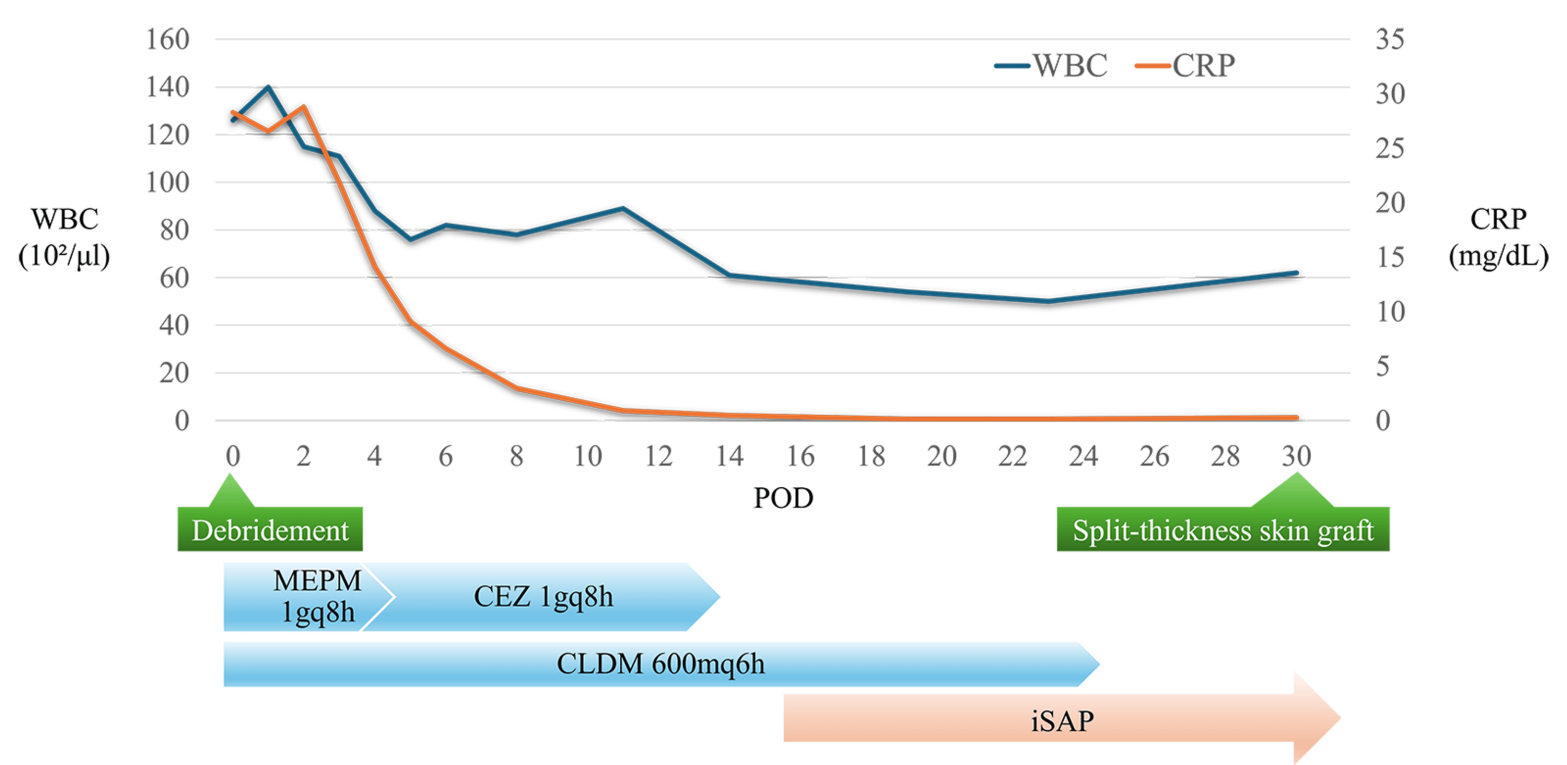
Clinical course after admission iSAP was initiated on day 17 post-debridement. The inflammatory response improved steadily, and wound closure by split-thickness skin graft was performed on day 31 post-debridement. iSAP: intra-soft tissue antibiotic perfusion; WBC: white blood cell; CRP: C-reactive protein; POD: postoperative day; MEPM: meropenem; CEZ: cefazolin; CLDM: clindamycin

**Figure 6:**
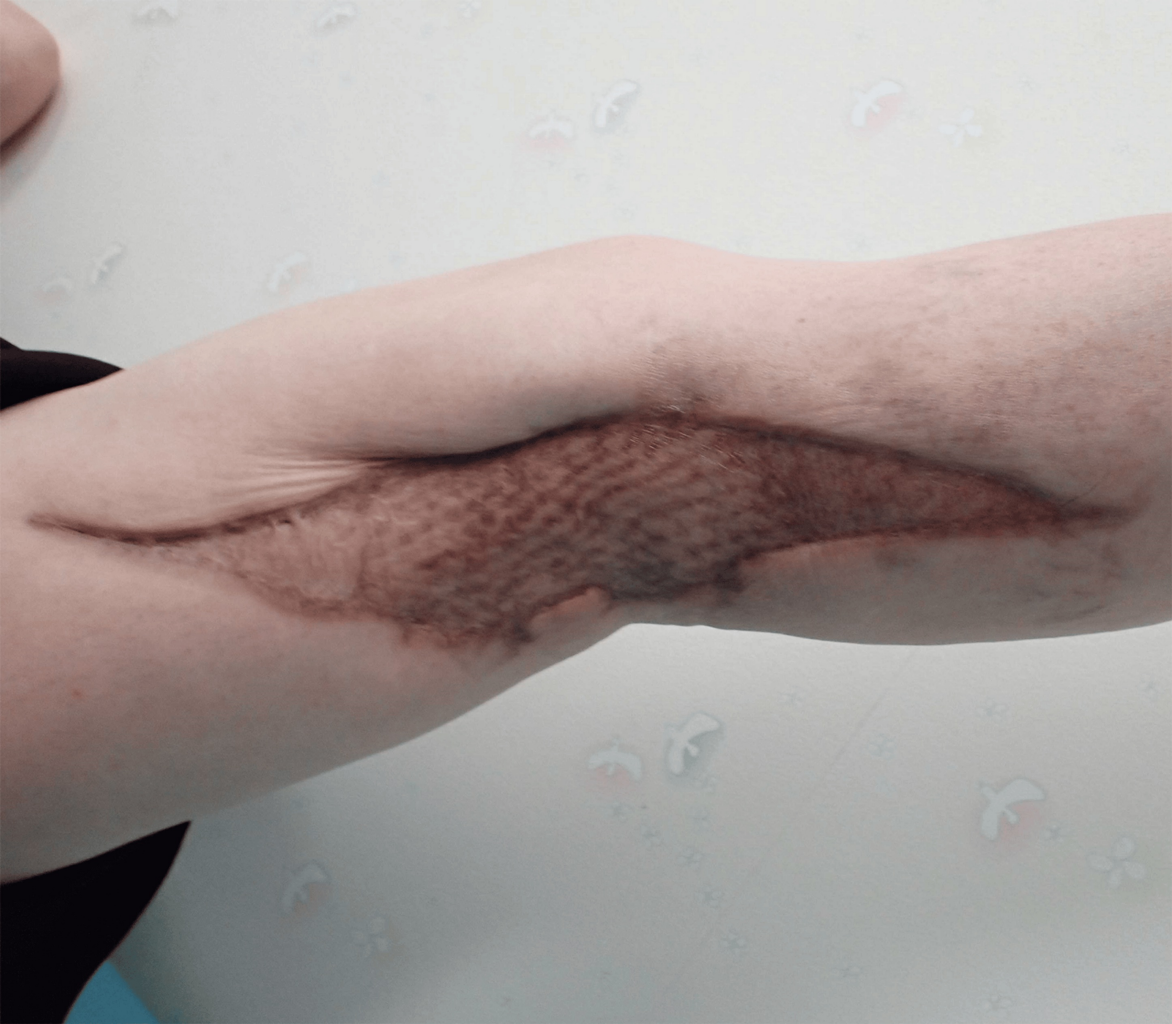
Photograph of the wound one year postoperatively Photograph of the wound one year postoperatively showing no recurrence of the ulcer.

Two weeks after the use of iSAP, wound closure was performed via a split-thickness skin graft under general anesthesia (Figure 4). NPWT for skin graft fixation was performed for one week and resulted in favorable wound healing. The patient was discharged on POD 45 with the ability to walk independently (Figure 5). One year postoperatively, there has been no recurrence of the ulcer (Figure 6).

Case 2

**Figure 7:**
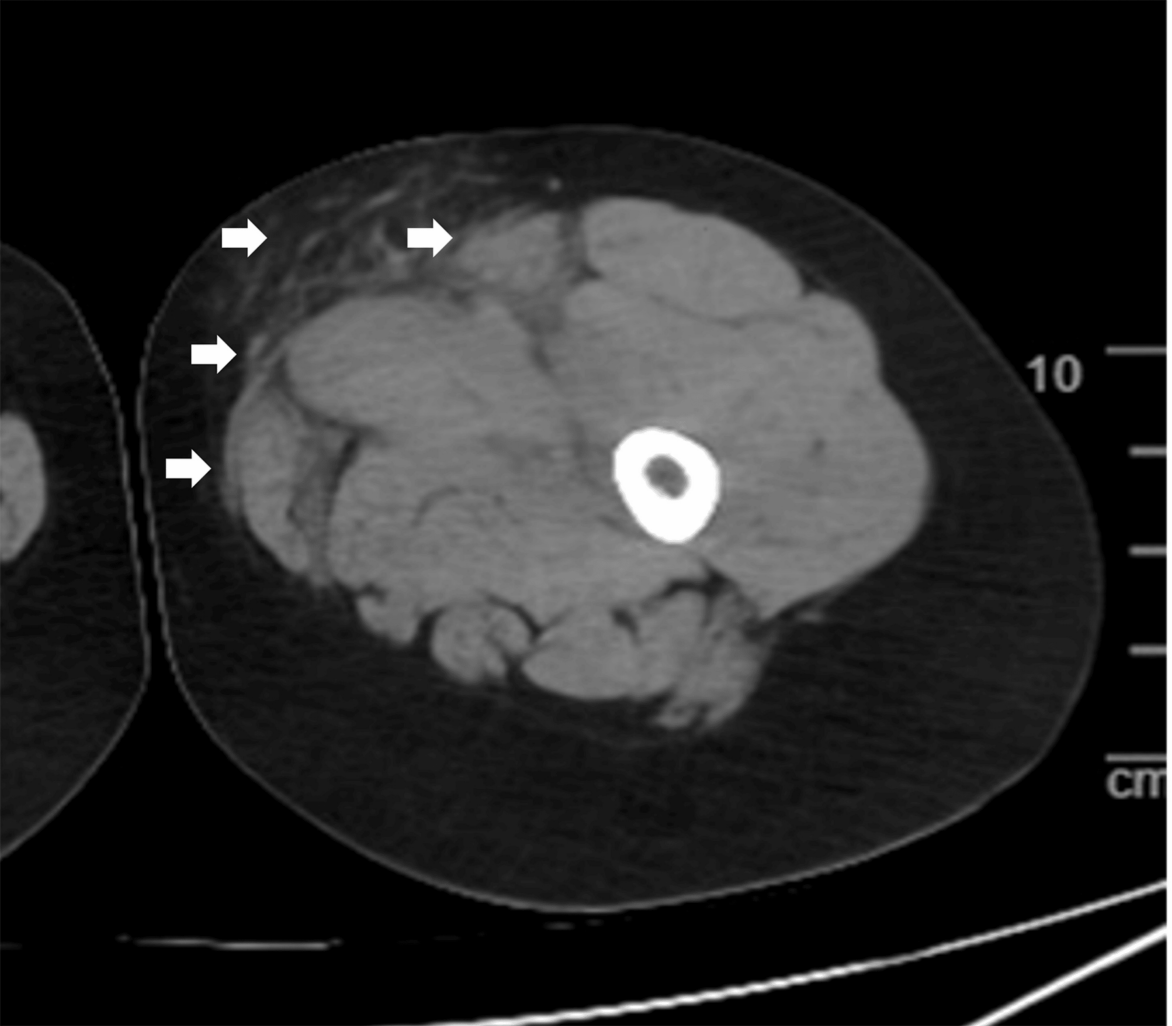
CT findings CT findings showing swelling of the sartorius muscle, gracilis muscle, and adductor muscle group, along with increased attenuation of the surrounding fat (white arrows). CT: computed tomography

The patient was a 23-year-old female without underlying medical conditions. She noticed pain in her left thigh three days prior and was emergently transported to our hospital due to worsening symptoms and fever. Upon arrival, her vital signs were as follows: BT 38.8°C, BP 120/80 mmHg, HR 120 beats/min, and SpO2 98% on room air. The patient tested positive for COVID-19 upon arrival. Erythema and pain were observed on the inner aspect of the left thigh. CT revealed swelling of the sartorius muscle, gracilis muscle, and adductor muscle group, along with increased attenuation of the surrounding fat (Figure 7). Blood tests showed WBC 26,800/μL, CRP 31.82 mg/dL, Hb 14.3 g/dL, Na 125.1 mmol/L, Cre 0.74 mg/dL, and Glu 126 mg/dL. The LRINEC score was 9.

**Figure 8:**
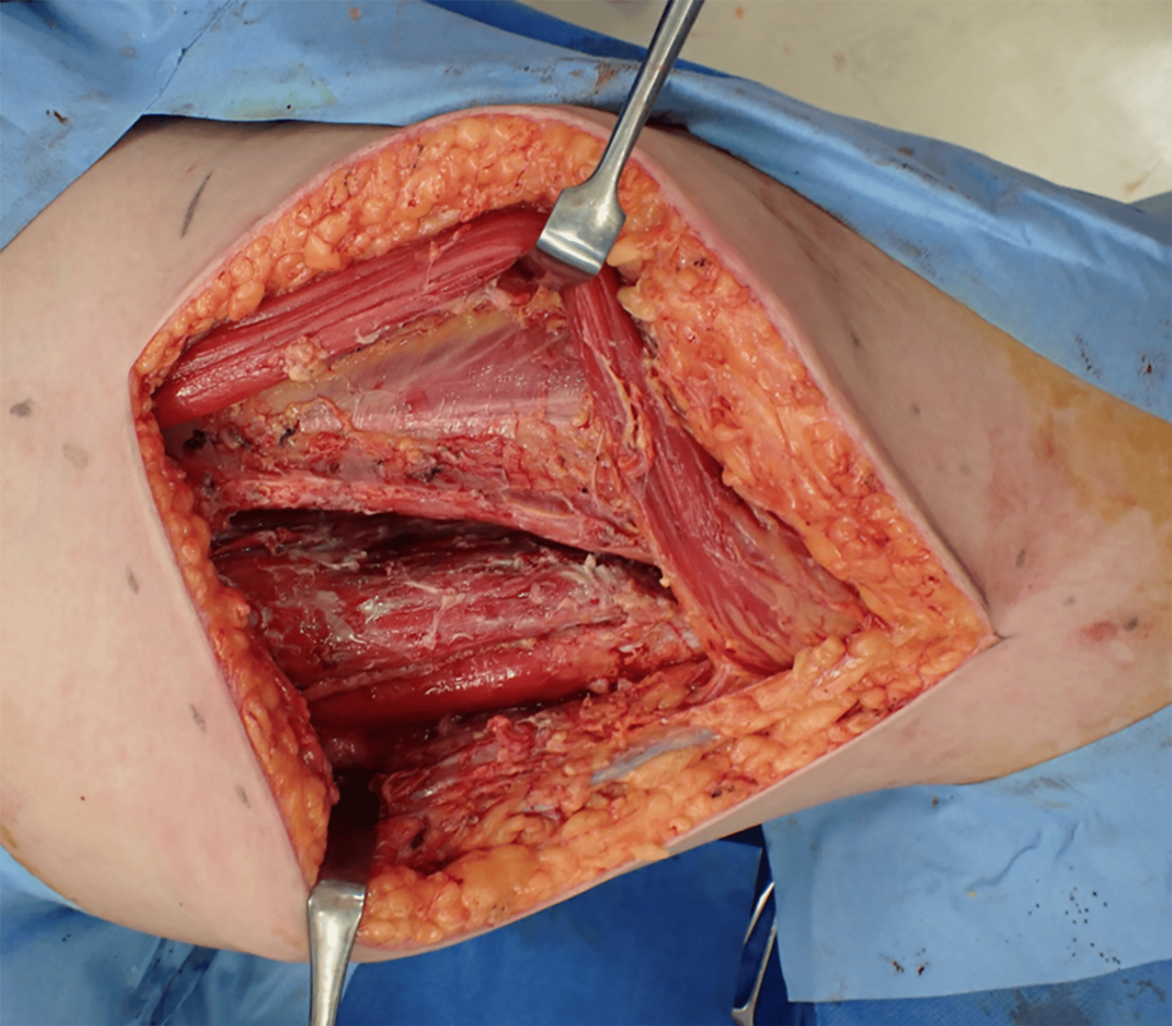
Intraoperative findings Intraoperative findings showing that the infection had spread along the adductor magnus muscle and the superficial femoral artery.

A diagnosis of NF was made and debridement was conducted under general anesthesia. Upon opening the compartment of the adductor muscles, copious purulent discharge was observed and was collected for wound culture analysis. The infection was noted to have spread along the adductor magnus muscle and the superficial femoral artery, necessitating complete release of the involved area (Figure 8). Antibiotics were initiated with MEPM 2 g/day and CLDM 2400 mg/day. The wound was left open and daily wound irrigation was performed. From POD 2, vancomycin 1 g/day was added. Bacterial cultures from two sites of the wound identified *Streptococcus pyogenes* and α-*Streptococcus* species. From POD 5, the antibiotic regimen was de-escalated to ampicillin 6 g/day and CLDM 1200 mg/day.

**Figure 9:**
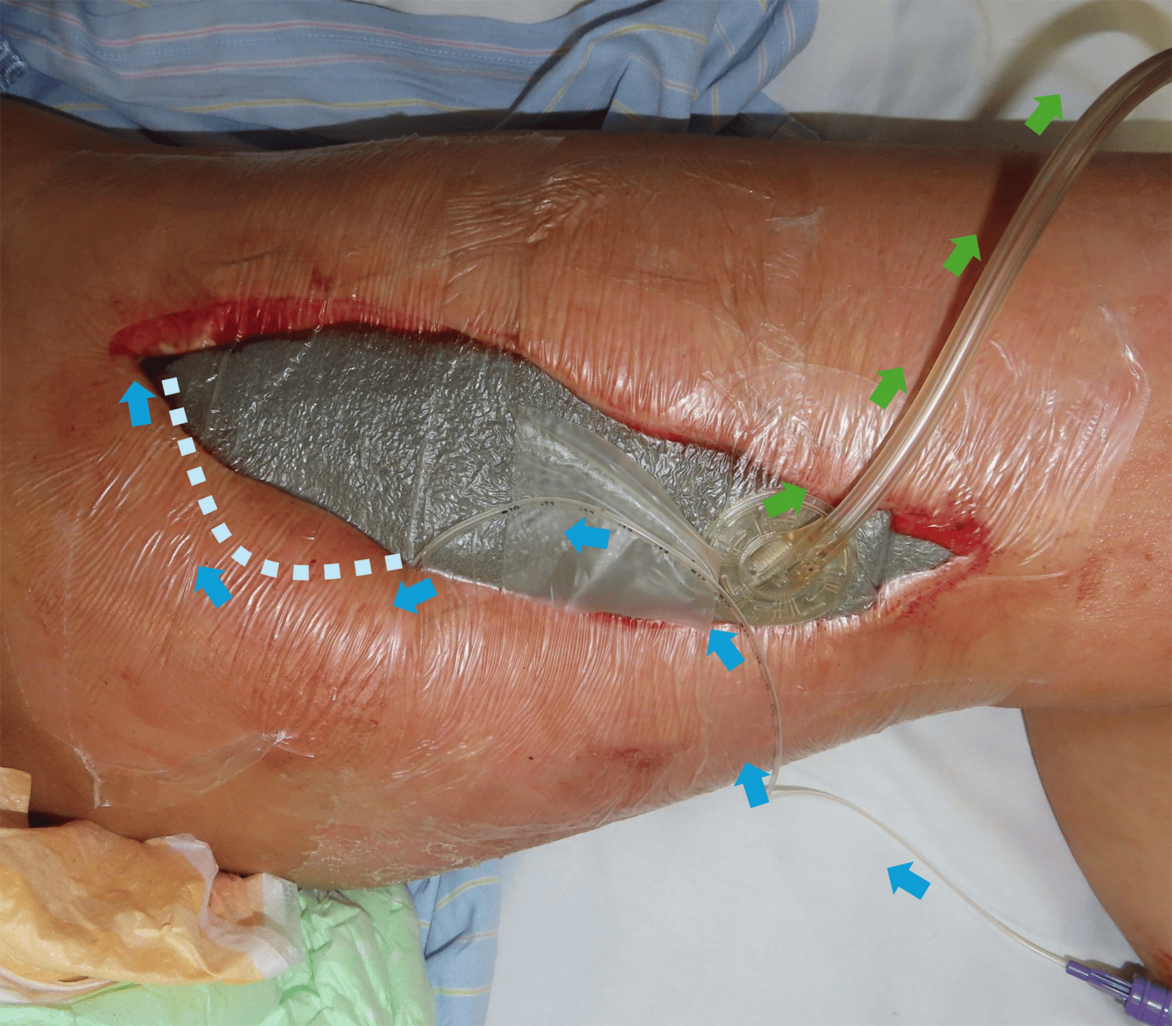
iSAP iSAP after debridement for necrotizing fasciitis. A gentamicin injection tube was positioned under the proximal thigh skin (blue arrows), and a suction tube was attached distally to the wound (green arrows). iSAP: intra-soft tissue antibiotic perfusion

iSAP was initiated on POD 7, after the confirmation of a certain decrease of necrotic tissue. A DACC-coated dressing (Sorbact® Compress, Abigo Medical AB, Sweden) was applied over the superficial femoral artery, and iSAP was performed. A gentamicin injection tube was positioned proximal to the adductor magnus muscle, and a suction tube was attached distally to the wound (Figure 9).

**Figure 10:**
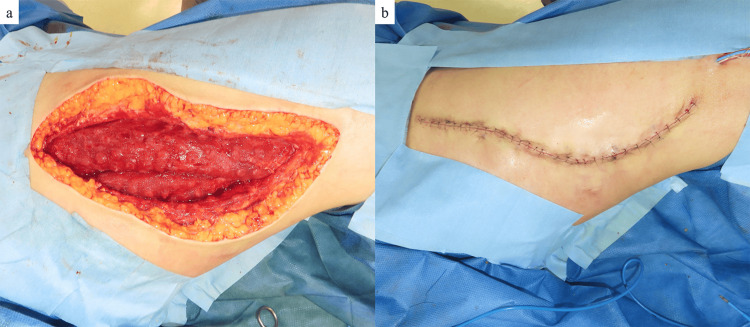
One week after the iSAP application The necrotic tissue had disappeared, and good granulation tissue growth was observed (a). Wound closure was then performed by simple closure (b). iSAP: intra-soft tissue antibiotic perfusion

**Figure 11:**
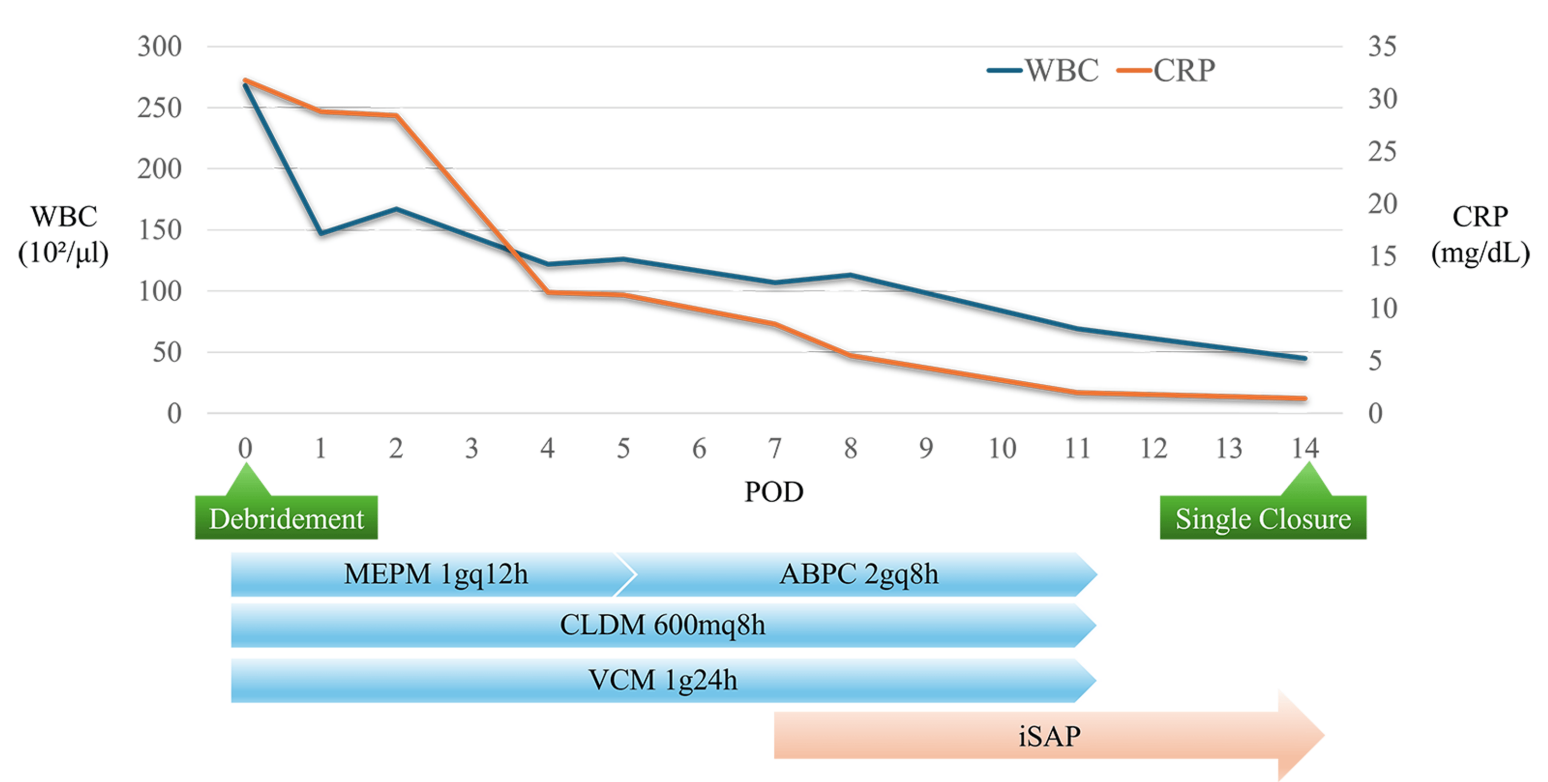
Clinical course after admission iSAP was initiated on day 7 post-debridement. The inflammatory response improved steadily, and wound closure by simple suturing was performed on day 14 post-debridement. iSAP: intra-soft tissue antibiotic perfusion; WBC: white blood cell; CRP: C-reactive protein; POD: postoperative day; MEPM: meropenem; CLDM: clindamycin

**Figure 12:**
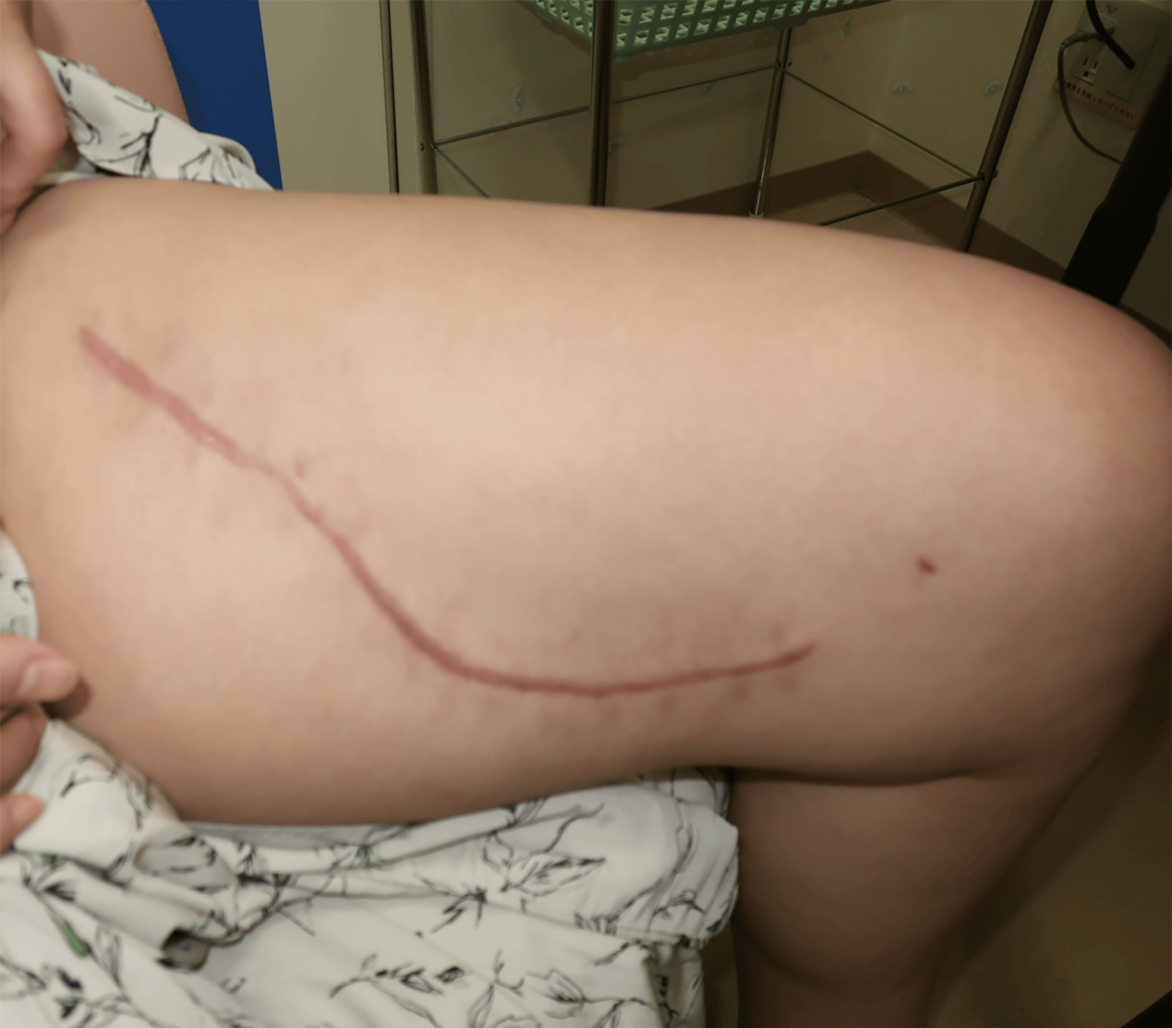
Photograph of the wound one year postoperatively Photograph of the wound one year postoperatively showing hypertrophic scarring, but no recurrence of the ulcer.

Foam exchange was performed twice a week. No further spread of infection occurred after the use of iSAP. Necrotic tissue was removed, and a marked improvement in the wound was noted; thus, antibiotics were discontinued on POD 11. One week after the start of iSAP, the wound was closed by simple closure on POD 14 (Figure 10). Postoperative recovery was uneventful, and the patient was discharged home on POD 20 (Figure 11). There has been no recurrence of the ulcer for six months postoperatively (Figure 12).

## Discussion

Bacteria that colonize an organism can form a biofilm, which may block the effects of antimicrobial agents and immune responses. Thus, bacteria in biofilms exhibit reduced susceptibility to antimicrobials, which contributes to the delayed healing of contaminated and chronic wounds [17]. In antimicrobial susceptibility tests, the effectiveness of agents is usually determined by the minimum inhibitory concentration (MIC). However, to counteract biofilm growth, a minimum biofilm eradication concentration (MBEC) that is 100-1,000 times greater than the MIC is necessary [18]. Achieving MBEC levels in the bloodstream via systemic administration would require doses that risk overdose, rendering such treatment impractical. iSAP facilitates the direct administration of antimicrobials at the infection site, allowing high local concentrations that avoid systemic side effects. Aminoglycosides such as gentamicin, for which the bactericidal activity depends on concentration, are recommended for CLAP, including iSAP [14-16].

The mutant selection window (MSW) defines the antibiotic concentration range between the MIC and the mutant prevention concentration (MPC), at which the growth of resistant mutants is prevented. Although resistant bacteria are typically induced within the MSW [19], iSAP maintains gentamicin levels above the MPC locally, thus reducing the likelihood of inducing resistant strains. However, gentamicin has potential side effects of ototoxicity and nephrotoxicity. To mitigate these risks, serum levels are monitored twice weekly, with an adjustment of the dosage if the level exceeds 2 µg/ml [14-16]. Junker et al. have shown that gentamicin at 2,000 µg/ml treats infected wounds effectively, while a concentration of 1,000 µg/ml inhibits cell proliferation [20]. In our cases, there were no increases in the serum level and no complications, and there were positive trends for wound healing and granulation.

NPWT is an effective method post-debridement for managing NF wounds [9-11], but the semi-closed environment created by NPWT poses an infection risk [12]. This highlights the importance of complete debridement and infection control prior to NPWT initiation. However, overly aggressive debridement can harm healthy tissue preservation, whereas prolonged conservative treatment increases both the patient burden and healthcare costs. iSAP offers a novel approach to combat infection in NF by enhancing infection control and accelerating wound healing by the infusion of high-concentration antibiotics locally using NPWT. iSAP may also reduce the need for prolonged hospital stays and additional surgeries, offering economic benefits to the healthcare system. Unlike NPWTi-d, in which wounds are intermittently irrigated with saline, iSAP focuses primarily on infection control through localized high-concentration antibiotic administration. Effective iSAP utilization requires initiation post-debridement and after the removal of a significant amount of necrotic tissue. We note that the small sample size in this study is a limitation, and thus, further clinical and basic studies are needed to refine the timing and duration for the initiation of iSAP.

## Conclusions

This case report underscores the promising role of iSAP as an adjunctive therapy in the management of NF post-debridement. Integration of iSAP into existing wound management protocols was shown to enhance infection control, complementing the benefits of NPWT. These findings suggest that iSAP could significantly improve outcomes for patients with NF. Further studies in more cases and with controlled designs are needed to substantiate these observations and refine the application of iSAP in the clinical practice for NF treatment.

## References

[1] Puvanendran R, Huey JC, Pasupathy S (2009). Necrotizing fasciitis. Can Fam Physician.

[2] Rajput A, Waseem Waseem, Samad A, Khanzada TW, Shaikh GM, Channa GA (2008). Mortality in necrotizing fasciitis. J Ayub Med Coll Abbottabad.

[3] Cheng NC, Su YM, Kuo YS, Tai HC, Tang YB (2008). Factors affecting the mortality of necrotizing fasciitis involving the upper extremities. Surg Today.

[4] Mok MY, Wong SY, Chan TM, Tang WM, Wong WS, Lau CS (2006). Necrotizing fasciitis in rheumatic diseases. Lupus.

[5] Golger A, Ching S, Goldsmith CH, Pennie RA, Bain JR (2007). Mortality in patients with necrotizing fasciitis. Plast Reconstr Surg.

[6] Anghel EL, Kim PJ (2016). Negative-pressure wound therapy: a comprehensive review of the evidence. Plast Reconstr Surg.

[7] Hideharu N, Takaya M, Masaru O, Satoshi Y (2023). Negative pressure wound therapy for skin ulceration and fistula after surgery for oral cancer. J Craniofac Surg Open.

[8] Hideharu N, Takaya M, Yasuko H (2023). Negative pressure wound therapy for inguinal lymphorrhea. Int J Surg Wound Care.

[9] de Paula FM, Pinheiro EA, Oliveira VM, Ferreira CM, Monreal MT, Rolan MD, Matos VT (2019). A case report of successful treatment of necrotizing fasciitis using negative pressure wound therapy. Medicine (Baltimore).

[10] Livingstone JP, Hasegawa IG, Murray P (2018). Utilizing negative pressure wound therapy with instillation and dwell time for extensive necrotizing fasciitis of the lower extremity: a case report. Cureus.

[11] Afzal M, Shakoor H, Afzal MT (2023). A case report of necrotizing fasciitis managed with the application of negative pressure wound therapy to achieve better wound closure. Cureus.

[12] Weed T, Ratliff C, Drake DB (2004). Quantifying bacterial bioburden during negative pressure wound therapy: does the wound VAC enhance bacterial clearance?. Ann Plast Surg.

[13] Faust E, Opoku-Agyeman JL, Behnam AB (2021). Use of negative-pressure wound therapy with instillation and dwell time: an overview. Plast Reconstr Surg.

[14] Himeno D, Matsuura Y, Maruo A, Ohtori S (2022). A novel treatment strategy using continuous local antibiotic perfusion: a case series study of a refractory infection caused by hypervirulent Klebsiella pneumoniae. J Orthop Sci.

[15] Maruo A, Oda T, Mineo R (2022). Continuous local antibiotic perfusion: a treatment strategy that allows implant retention in fracture-related infections. J Orthop Surg (Hong Kong).

[16] Kitano D, Sakurai A, Kuwazuru K, Kitagawa H, Taniguchi T, Takahara S (2023). Intra-soft tissue and intramedullary antibiotic perfusion in combination with negative pressure wound therapy. J Wound Care.

[17] Hathroubi S, Mekni MA, Domenico P, Nguyen D, Jacques M (2017). Biofilms: microbial shelters against antibiotics. Microb Drug Resist.

[18] Ceri H, Olson ME, Stremick C, Read RR, Morck D, Buret A (1999). The Calgary Biofilm Device: new technology for rapid determination of antibiotic susceptibilities of bacterial biofilms. J Clin Microbiol.

[19] Drlica K (2003). The mutant selection window and antimicrobial resistance. J Antimicrob Chemother.

[20] Junker JP, Lee CC, Samaan S (2015). Topical delivery of ultrahigh concentrations of gentamicin is highly effective in reducing bacterial levels in infected porcine full-thickness wounds. Plast Reconstr Surg.

